# *In Vivo* Inhibition of MicroRNA-326 in a NOD.H-2^h4^ Mouse Model of Autoimmune Thyroiditis

**DOI:** 10.3389/fimmu.2021.620916

**Published:** 2021-06-01

**Authors:** Na Zhao, Zhenzhen Wang, Xuejiao Cui, Shuo Wang, Chenling Fan, Yushu Li, Zhongyan Shan, Weiping Teng

**Affiliations:** Department of Endocrinology and Metabolism, Institute of Endocrinology, NHC Key Laboratory of Diagnosis and Treatment of Thyroid Diseases, Liaoning Provincial Key Laboratory of Endocrine Diseases, The First Affiliated Hospital of China Medical University, Shenyang, China

**Keywords:** autoimmune thyroiditis, miR-326, Th17/Treg, Ets-1, NOD.H-2^h4^ mice, *in vivo*

## Abstract

**Background:**

Previous studies reported that various miRNAs participate in autoimmune diseases, but the potential regulatory mechanism of miRNAs in autoimmune thyroiditis (AIT) needs further exploration.

**Objective:**

This study aimed to further verify that miR-326 contributes to AIT by regulating Th17/Treg balance through Ets-1 using lentiviral gene delivery through tail vein and thyroid injection in NOD.H-2^h4^ mice.

**Materials and Methods:**

Five-week-old NOD.H-2^h4^ mice were divided randomly into tail vein and thyroid injection groups, and each received either mmu-miR-326 sponge (LV-sponge) or lentiviral vector control. Mice were divided for tail vein injection: the therapeutic LV-ctrl, therapeutic LV-sponge, prophylactic LV-ctrl, and prophylactic LV-sponge groups. The control group was fed high-iodine water without vein injection. The thyroid infiltration of lymphocytes and serum TgAb value were investigated by thyroid hematoxylin and eosin (HE) staining and ELISA, respectively. Ets-1 and lymphocyte counts were measured by RT-PCR, western blotting, and flow cytometry. The thyroid CD4^+^IL-17a^+^ cells and CD4^+^Ets-1^+^ cells were detected by immunofluorescence, and the serum cytokines were tested by ELISA.

**Results:**

In the tail vein injection groups, the thyroid inflammatory score and serum TgAb titer were significantly lower in the LV-sponge groups than in the control and LV-ctrl groups while Ets-1 protein expression in mouse spleens was increased in the LV-sponge groups. Moreover, Th17/Treg ratio declined in the LV-sponge group and decreased significantly in the prophylactic LV-sponge group (*P* = 0.036) tested by flow cytometry. Immunofluorescence showed that, in LV-sponge groups, CD4^+^IL-17a^+^ cells were decreased significantly (*P* = 0.001), while CD4^+^Ets-1^+^ cells were increased significantly in the LV-sponge group (*P* = 0.029). The serum IL-17/IL-10 was decreased significantly in the LV-sponge group (*P* < 0.05). In the thyroid injection groups, the thyroid inflammatory score and serum TgAb titer in the LV-sponge group decreased significantly compared with those in the LV-ctrl group (*P* < 0.05). In addition, in LV-sponge groups, CD4^+^IL-17a^+^ cells were decreased, while CD4^+^Ets-1^+^ cells were increased significantly in the inhibition group evaluated by immunofluorescence. Moreover, tail vein injection of LV-sponge resulted in much lower TgAb levels in thyroiditis compared with thyroid injection.

**Conclusion:**

MiR-326 targeted therapy may be a promising approach for AIT. In addition, tail vein injection may achieve a better intervention effect than thyroid injection.

## Introduction

Autoimmune thyroiditis (AIT), a type of autoimmune thyroid disease (AITD), mainly includes Hashimoto’s thyroiditis (HT), atrophy thyroiditis, and Riedel thyroiditis. The thyroid glands of patients with AIT undergo lymphocyte infiltration and thyroid follicular destruction. Circulating serum autoantibodies such as thyroid peroxidase antibody (TPOAb) and thyroglobulin antibody (TgAb) in HT patients lead to thyroid atrophy or fibrosis accompanied by hypothyroidism (1, 2). It has been reported that environmental factors trigger the occurrence of AIT in a genetic background. According to experimental studies using animal models with genetic susceptibility and epidemiological studies, the incidence of AIT was increased by high iodine intake (3–6). Nevertheless, the specific mechanism of AIT remains unclear. AIT is often interrelated with other autoimmune diseases, such as rheumatoid arthritis (RA), systemic lupus erythematosus (SLE), and type I diabetes (T1D) (7). Elucidating AIT pathogenesis could benefit AIT diagnosis and prevention.

Through antigen recognition and the presence of cytokines, naïve CD4^+^ T cells differentiated into Th1 (mainly synthesized IFN-*γ*), Th2 (mainly synthesized IL-4), Th17, and regulatory T (Treg) cells, participate in the occurrence and progression of autoimmune diseases. Th17 cells producing IL-17 promote inflammation, while Treg cells producing cytokines IL-10 anti-inflammatory inhibit immune responses. Th17/Treg balance plays a vital role in many autoimmune diseases. Numerous studies suggest that the Th17/Treg balance resides in the core of pathogenic mechanisms driving autoimmune diseases and can be used as an important indicator of disease status (8–13). The breakdown of the Th17/Treg ratio participated in AITD, which was thought to contribute to the disruption of the Th1/Th2 balance (14, 15). A variety of mechanisms related to Treg and Th17 participate in AITD (11). For example, the Ets-1 protein, an Ets family transcription factor with a unique Ets domain that could bind to different DNA sequences, could act as a negative regulator of Th17 cells (16). Meanwhile, Ets-1 plays an important role in the suppressive activity of CD4^+^CD25^+^ Treg cells and stable expression of Foxp3 (17). The present study will further evaluate lymphocyte subsets and their regulatory mechanisms in an autoimmune thyroiditis mice model.

NOD.H-2^h4^ mice were obtained by hybridizing B10.A (4R) and NOD mice and then backcrossing with MHC haplotype (H-2K) expression of B10.A (4R) offspring to NOD mice (18, 19). H-2K mice are genetically susceptible to autoimmune thyroid diseases (20). This mouse model had lifelong SAT characterized by intrathyroidal lymphocyte infiltration and increased TgAbs. Furthermore, overdose iodide intake promoted SAT incidence and severity in NOD.H2^h4^ mice, resulting as an ideal AIT experimental animal model (19, 21).

MicroRNAs (miRNAs) are a class of small, endogenous, single-stranded, non-coding RNAs that usually contain 18–24 nucleotides. Typically, miRNAs inhibit gene expression at the posttranscriptional level through specific bonds with the 3′-UTR of target mRNAs. In our previous study, miR-326 participated in Th17 regulation by targeting Ets-1 protein *in vitro (* 22 *).* This study further verified that miR-326 participated in AIT by regulating Ets-1 protein to regulate Th17/Treg balance through *in vivo* intervention.

## Materials and Methods

### Mice and Groups

NOD.H-2^h4^ mice were obtained from the Jackson Laboratory (Bar Harbor, ME, USA) and were bred and kept in a pathogen-free environment at the Animal Experiment Center of China Medical University. All protocols for animal care and experiments were in accordance with the Guidelines for Animal Experimentation and were approved by the Animal Ethics Committee of China Medical University. Two kinds of lentivirus (mmu-mir-326 sponge, LV-sponge, 1 × 10^9^ TU/ml) and lentivirus-specific control (LV-ctrl) synthesized by HANBIO (Shanghai, China) were used for tail vein injection and local thyroid injection in mice. Sponge is a miR-326-specific decoy target that can bind to miR-326 to inhibit the activity of endogenous miR-326 without affecting its transcripts. NOD.H-2^h4^ mice (5 weeks old) were randomly divided into five groups and given high-iodine water that contained 0.05% sodium iodide (NaI) (approximately 1,000 times the normal daily iodine intake) for 12 weeks. The five groups were the control group, therapeutic lentiviral vector control group (therapeutic LV-ctrl), therapeutic lentiviral vector inhibitor of mir-326 group (therapeutic LV-sponge), prophylactic lentiviral vector control group (prophylactic LV-ctrl), and prophylactic lentiviral vector inhibitor of mir-326 group (prophylactic LV-sponge group). Except for the control group, the other four groups were treated with lentivirus tail vein intervention. In the therapeutic groups, the starting time of mouse tail vein injection was followed by 6 weeks of iodine water feeding, while the beginning time of tail vein injection was the same as that of iodine water feeding in the prophylactic group. Each mouse was injected two times, 1 × 10^7^ TU/each time, with an interval of two weeks. Mice were sacrificed by iodine feeding for 12 weeks. In the intrathyroid injection experiments, 5-week-old NOD.H-2^h4^ mice were divided into the control group (LV-ctrl) and LV-sponge groups. After skin preparation of the mouse neck, the thyroid gland was exposed by longitudinal incision. Lentivirus was injected into the thyroid bilaterally once at 0.5 × 10^7^ TU per lobe at the beginning of high-iodine water feeding, and the mice were sacrificed after iodine feeding for 10 weeks.

### RT-qPCR Analysis

To determine the level of mRNA, RNA was reverse transcribed into mRNA by PrimeScriptTM RT Reagent Kit 036A (TaKaRa) and quantified by SYBR^®^ Premix Ex TaqTM II 820A (TaKaRa). Data normalization of each sample was performed according to the GAPDH level. RT-qPCR analysis was carried out with a LightCycler 480 system. The primer sequences (synthesized by Sangon Biotech, Shanghai) are listed in **Table 1**.

**Table 1:** mRNA sequences of primers used for RT-qPCR.

Gene	Primer sequences
Mouse Ets-1-F:	5′-GAGCAGCCAGTCATCCTTCAACAG-3′
Mouse Ets-1-R:	5′-CAGCACGGTCACGCACATAGTC-3′
Mouse IL-17a-F:	5′-TGATGCTGTTGCTGCTGCTGAG-3′
Mouse IL-17a-R:	5′-TGGAACGGTTGAGGTAGTCTGAGG-3′
Mouse IL-22-F:	5′-TCCAACTTCCAGCAGCCATACATC-3′
Mouse IL-22-R:	5′-GCACTGATCCTTAGCACTGACTCC-3′
Mouse Rorgt-F:	5′-GTCCAGACAGCCACTGCATTCC-3′
Mouse Rorgt-R:	5′-TGCCGTAGAAGGTCCTCCAGTC-3′
Mouse Rora-F:	5′-CCACCTACTCCTGTCCTCGTCAG-3′
Mouse Rora-R:	5′-CTTCTGCACCTCGGCGTACAAG-3′
Mouse Irf4-F:	5′-GGCAAGCAGGACTACAATCGTGAG-3′
Mouse Irf4-R:	5′-TGGCTCCTCTCGACCAATTCCTC-3′
Mouse Hif1a-F:	5′-CCACCACAACTGCCACCACTG-3′
Mouse Hif1a-R:	5′-TGCCACTGTATGCTGATGCCTTAG-3′
CCR6-F:	5′-TGTGAGCCACGGTACAGGTCTG-3′
CCR6-R:	5′-CTCGGATTGCTCTGTGCCTCTTG-3′
Mouse IL-23r-F:	5′-ATGTGCTCTTCAGATGGTGTCACG-3′
Mouse IL-23r-R:	5′-AAGATTCCTTGGTCGGCAGTGC-3′

F, forward; R, reverse.

### Flow Cytometry Analysis of Lymphocytes

For staining T/B/cytotoxic T cells (Tc)/helper T cells (Th), spleen mononuclear cells were obtained and incubated with rat anti-mouse CD16/CD32 (Fc-block) to block Fc-receptor binding. Then, anti-mouse CD3-FITC, anti-mouse CD4-PerCP, anti-mouse CD8a-PE, and anti-mouse B220-APC were added and incubated at room temperature for 20 min to test extracellular proteins. For Th1/Th2/Th17/Treg cell staining, spleen mononuclear cells were obtained and incubated with a cell activation cocktail (with Brefeldin A) at 37°C for 5 h. Then, the cells were incubated with Fc-block at room temperature for 10 min to avoid non-specific binding. To detect extracellular proteins, anti-mouse CD4-PerCP and anti-mouse CD25-APC were added before intracellular IL-17, IL- 4, and IFN-*γ* antibody staining and were incubated at room temperature for 20 min. For intranuclear staining after extracellular antibodies were added, the True-Nuclear™ Transcription Factor Buffer Set was applied before and after anti-mouse Foxp3-PE antibody incubating for 20 min at room temperature in the dark. The lymphocyte cells were tested and analyzed with FACScan Flow Cytometry and WinMDI2.9. Each sample was set to analyze 20,000 cells. All the reagents were purchased from BioLegend or BD, USA.

### SAT Induction and Evaluation

Mouse thyroid paraffin sections were collected for hematoxylin and eosin (HE) staining. The SAT mice exhibited thyroid lymphocyte infiltration and follicle expansion and lesions. The severity of lymphocyte infiltration in thyroiditis was rated by the collective areas of inflammatory lymphocyte cells as previously described (23): 0, no infiltration; 1, 1–10% infiltration; 2, 10–30% infiltration; 3, 30–50% infiltration; 4, greater than 50% infiltration. The thyroiditis score was expressed as the mean of at least three non-consecutive sections of thyroid tissue.

### ELISA Analysis of TgAbs

The level of serum mTg-specific IgG antibody was detected by enzyme-linked immunosorbent assay (ELISA) as previously reported (22–24). The serum from each individual mouse was diluted 1:200 with PBS. Peroxidase-labeled goat anti-mouse IgG diluted 1:5,000 in PBS (Sigma, USA) was added. A microplate reader (Bio-Rad 680, US) was used to read optical density values at 450 nm.

### ELISA Analysis of Serum Cytokine

Mice serum cytokines IFN-*γ*, IL-4, IL-17a, and IL-10 were analyzed using Luminex Mouse Discovery Assay (4-Plex, R&D Systems, USA). All samples were processed according to the manufacturer’s instructions.

### Western Blot Analysis of Ets-1 Protein

The mouse spleen proteins were extracted by a protein extraction kit (Keygen Biotech, Nanjing, China), and then the extracted supernatant was quantified with a BCA Protein Concentration Assay Kit (Beyotime, Shanghai, China). The specific western blot analysis procedures were performed according to a previous study (22). The primary antibodies were anti-ETS-1 antibody (1:1,000 dilution; Cell Signaling Technology) and *β*-actin (1:5,000; Proteintech). Peroxidase-conjugated goat anti-mouse IgG (1:5,000 dilution; Proteintech) was used as the secondary antibody.

### Immunofluorescence Analysis

Immunofluorescence assay was performed on frozen sections of thyroid tissue. Briefly, after 1% BSA added for preventing nonspecific binding, 5-μm sections of thyroid tissue was incubated by CD4^+^ (Biolegend), anti-IL-17a antibody (Affinity) and Ets-1 antibody (Affinity) as primary antibodies according to the manufacturer’s instructions. Then secondary antibodies were added. Under high-power (HP) microscope, three view fields of each mouse thyroid section were randomly selected to count the number of CD4^+^Ets-1^+^ cells CD4^+^IL-17^+^cells.

### Statistical Analysis

Data analyses were conducted in SPSS 22.0. Normally distributed data are presented as the mean ± standard deviation, and non-normally distributed data are expressed as the median (quartile). The distribution between the two groups was compared by independent sample t test or rank sum test, and *P <*0.05 was statistically significant.

## Results

### Inhibition of miR-326 by Lentivirus Injection in the Tail Vein

#### T cell and B Cell Subsets in NOD.H-2^h4^ Mice

**Figure 1:**
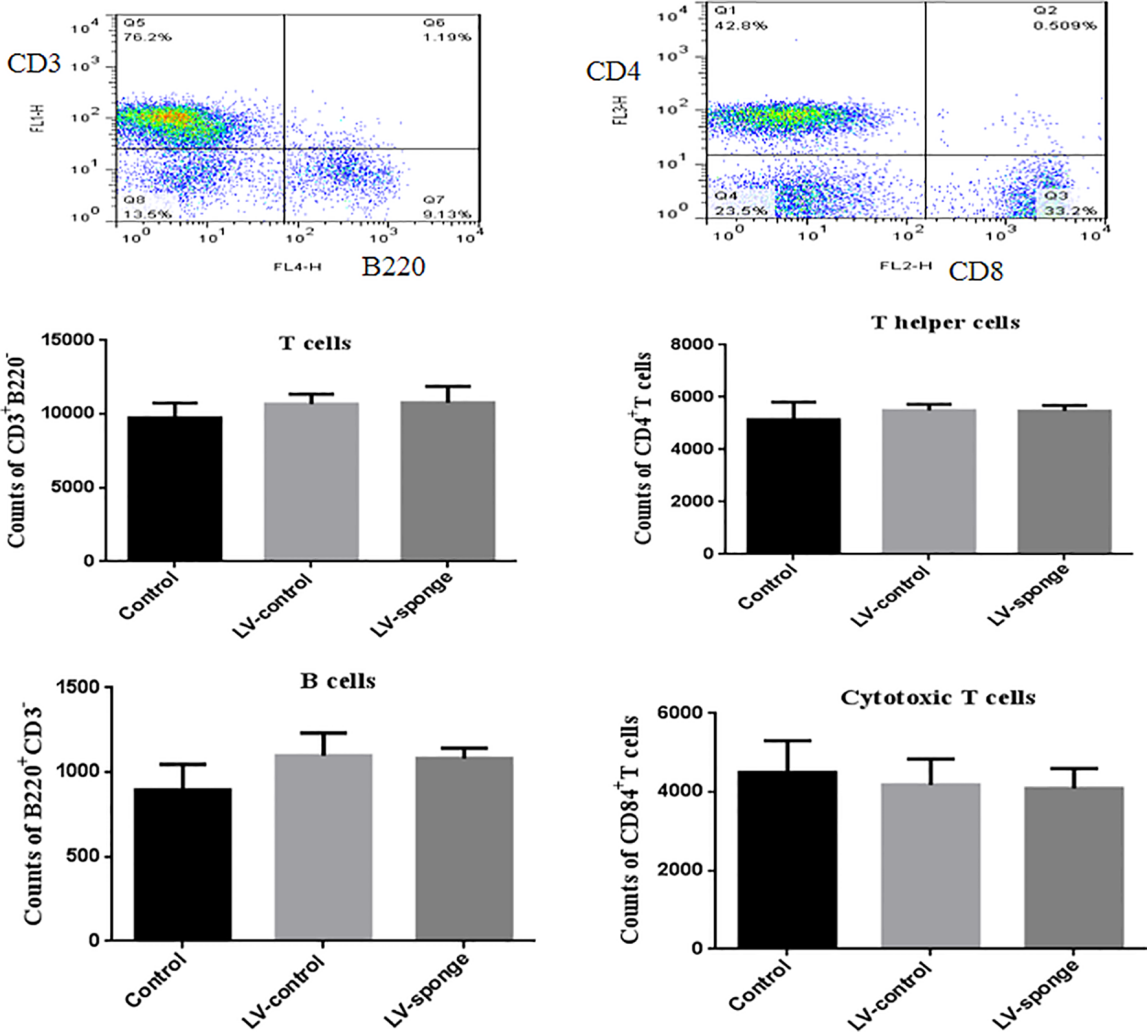
The balance of T cells, B cells, helper T cells (Th), and cytotoxic T cells (Tcs) was not affected by *in vivo* intervention. Flow cytometry analysis was used to assess the counts of T cells, B cells, Th, and Tc cells in LV-sponge groups, control group, and LV-control groups, N = 6–10 mice/group.

Flow cytometry analysis was used to test whether T and B cell balance was affected by miR-326 intervention. The results showed that the counts of T cells, B cells, cytotoxic T (Tc) cells, and helper T (Th) cells in LV-sponge groups were not different from those in the control group and LV-ctrl groups, suggesting that the *in vivo* intervention by lentivirus had no effect on the balances of T and B cell groups and Th and Tc cell subgroups in mice (**Figure 1**).

#### Lymphocyte Infiltration Inflammatory Score of Thyroid Glands and TgAb Titers in NOD.H-2^h4^ Mice

**Figure 2:**
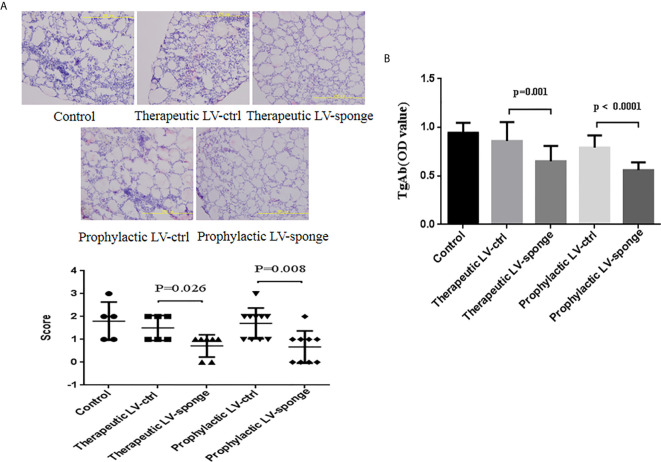
Lymphocyte infiltration inflammatory score of thyroid glands and TgAb titers in NOD.H-2h4 mice was decreased in LV-sponge groups. **(A)** Thyroid inflammation was determined according to the lymphocyte infiltration area using HE staining of frozen sections of the thyroid gland, 200×. **(B)** TgAb titers were determined by ELISA, N = 12/group.

Thyroid inflammation was determined according to the lymphocyte infiltration area using HE staining of frozen sections of the thyroid gland. Compared with LV-ctrl groups, we found that the scoring of thyroid inflammation in LV-sponge groups was decreased significantly (**Figure 2A**, *P* < 0.05). Consistent with the inflammatory score of the thyroid, the serum TgAb titers of the LV-sponge groups were significantly lower than those of the LV-ctrl groups (*P* < 0.01), and the TgAb level of the prophylactic LV-sponge group was significantly lower, as shown in **Figure 2B**.

#### Ets-1 mRNA Level and Protein Expression in the Spleen of NOD.H-2^h4^ Mice

**Figure 3:**
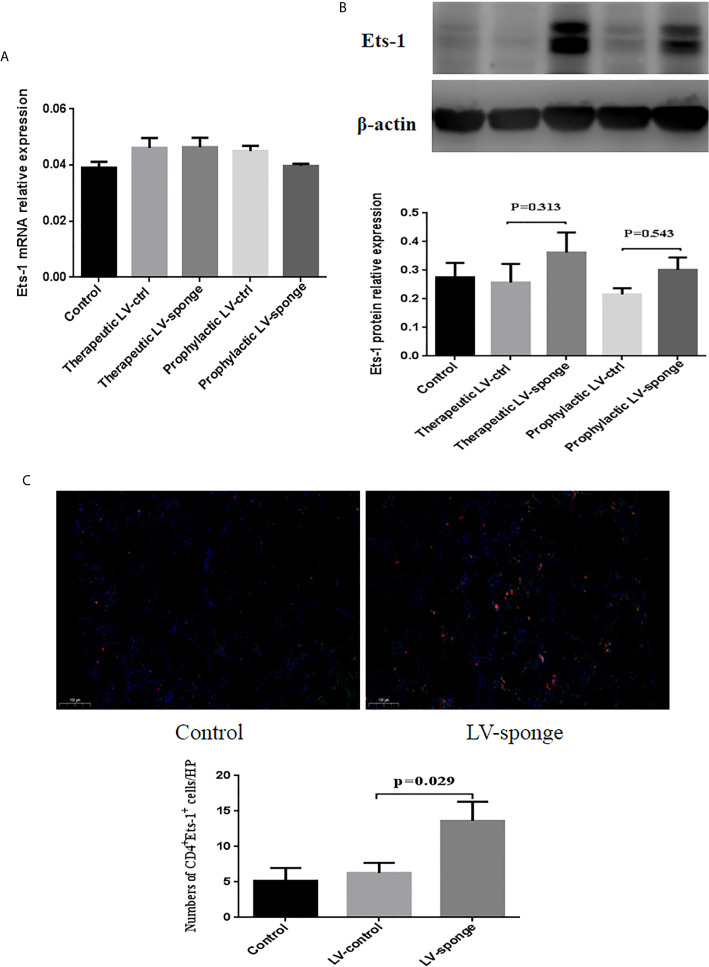
Ets-1 protein expression was increased in the spleens of NOD.H-2h4 mice in the LV-sponge groups. **(A)** RT-PCR was used to detect the levels of Ets-1 mRNA in the spleens of the five groups. **(B)** The relative expression of Ets-1 protein in the spleen was detected by western blot. **(C)** Immunofluorescence staining of CD4^+^Ets-1^+^ cells in thyroid sections. DAPI, blue; CD4^+^ Ets-1^+^ cells are shown in cyan in the nucleus (CD4^+^, red; Ets-1^+^, green). Under high-power (HP) microscope, three view fields of thyroid section were randomly selected to count the number of CD4^+^Ets-1^+^ cells. Scale bar, 100 μm.

RT-PCR was performed to measure Ets-1 mRNA levels in the spleen, and we found no significant difference among the five groups (**Figure 3A**). The expression of Ets-1 protein in the spleen was determined using western blot and compared with that in the LV-ctrl group; Ets-1 protein expression in the LV-sponge groups was increased. The difference was not significant (**Figure 3B**, *P* > 0.05). We further explored the expression of CD4^+^Ets-1^+^ cells in thyroid sections. We found that CD4^+^Ets-1^+^ cells were increased significantly in LV-sponge group (**Figure 3C**, *P* = 0.029).

#### CD4^+^ T Subset Expression in the Spleen of NOD.H-2^h4^ Mice

**Figure 4:**
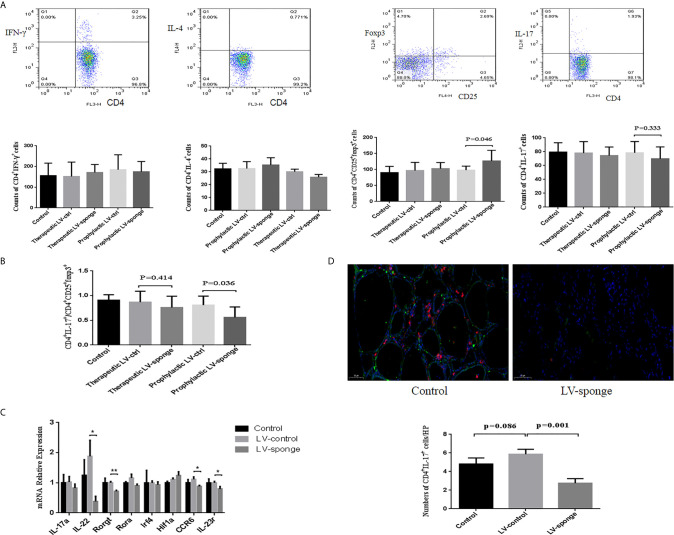
The counts of Th17 cells were decreased in the LV-sponge groups, while Treg cells were increased. **(A)** The counts of Th1, Th2, Th17, and Treg cells in the spleen assessed by flow cytometry at 12 weeks of high iodine water feeding. CD4^+^IFN-*γ* ^+^, CD4^+^IL-4^+^, CD4^+^IL-7^+^, and CD4^+^CD25^+^Foxp3^+^ T cells represented Th1, Th2, Th17, and Treg cells, respectively. **(B)** The counts of CD4^+^IL-7^+^/CD4^+^CD25^+^Foxp3^+^ (Th17/Treg) decreased significantly in the prophylactic LV-sponge group. **(C)** RT-PCR was used to evaluate the levels of cytokines, transcription factors, and surface receptors of Th17 cells (*P < 0.05; **P < 0.01). **(D)** Immunofluorescence staining of CD4^+^IL-17^+^ cells in sections of the thyroid. The CD4^+^ IL-17^+^ cells are shown in orange (CD4^+^, red; IL-17^+^, green). Under high-power (HP) microscope, three view fields of thyroid section were randomly selected to count the number of CD4^+^IL-17^+^ cells. Scale bar, 50 μm.

Flow cytometry was used to detect the counts of CD4^+^ T subset of lymphocytes. We found that there was no significant difference between Th1 and Th2 lymphocytes in the five groups. Compared with the LV-ctrl groups, the counts of Th17 cells decreased in the LV-sponge groups, but there was no significant difference compared with LV-ctrl groups. The counts of Treg cells increased in the LV-sponge group and increased significantly in the prophylactic LV-sponge group (*P* < 0.05). The Th17/Treg ratio decreased significantly in the prophylactic LV-sponge group (*P* = 0.036), **Figures 4A, B**. What’s more, immunofluorescence assay of thyroid tissue showed that, compared with control group, CD4^+^IL-17a^+^ cells were decreased significantly in LV-sponge groups (**Figure 4D**, *P* = 0.001). These results suggested that after inhibiting the activity of miR-326 *in vivo*, the Th17 cells promoting inflammation was decreased, and the percentage of Treg cells inhibiting inflammation was increased. Additionally, further RT-PCR results showed that after miR-326 inhibition *in vivo*, the cytokine IL-22, transcription factor Rorgt, and surface receptors CCR6 and IL-23r, which are related to Th17 cells, decreased significantly in the LV-sponge group (**Figure 4C**), while IL-17a and Rora showed a downward trend with no significant difference.

#### The Serum Cytokine Levels of NOD.H-2^h4^ Mice

**Figure 5:**
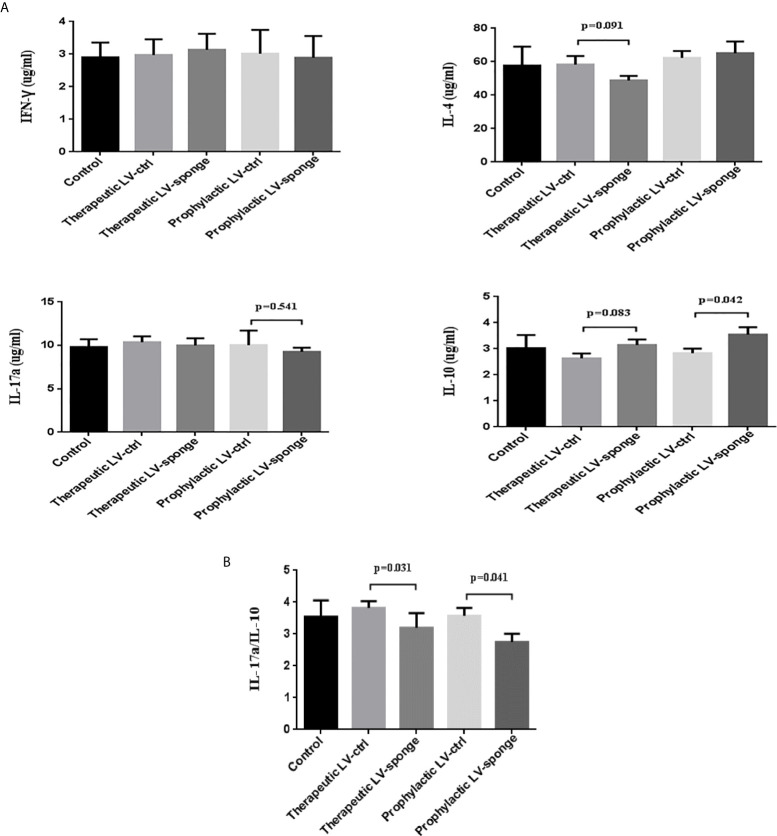
IL-17a/IL-10 were decreased significantly in LV-sponge group. **(A)** The serum cytokine secretion of IFN-*γ*, IL-4, IL-17a, and IL-10 was detected with ELISA. **(B)** The ratios of IL-17a/IL-10 were further evaluated in the five groups.

To evaluate the cytokine secretion intensities of IFN-*γ*, IL-4, IL-17a, and IL-10, we carried out ELISA experiment. We found that there was a downward trend of the level of IL-17a in the prophylactic LV-sponge group, while IL-10 level was increased significantly (*P* < 0.05). What’s more, IL-17a/IL-10 ratio was decreased significantly in LV-sponge group (**Figures 5A, B**, *P* < 0.05).

#### Inhibition of miR-326 by Lentivirus Injection Into the Thyroid

**Figure 6:**
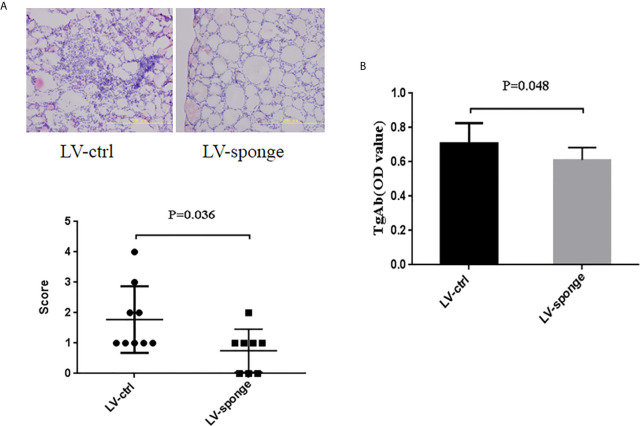
Evaluation of thyroiditis level by lentivirus thyroid injection inhibiting miR-326 activity. **(A, B)** The thyroiditis score and TgAb titers of NOD.H-2h4 mice in the LV-sponge group were significantly lower than those in the LV-ctrl group, N = 9–12 mice/group.

**Figure 7:**
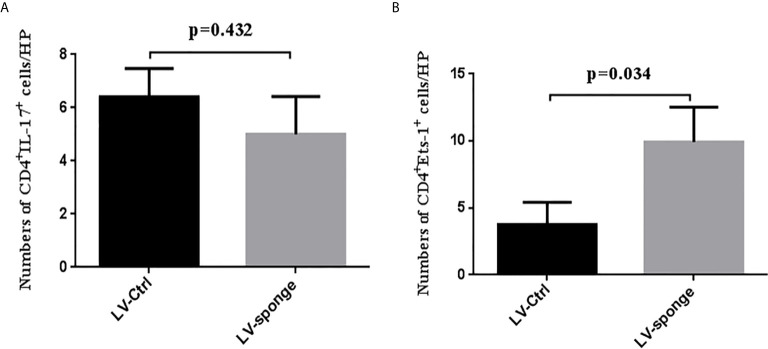
Immunofluorescence staining of CD4^+^IL-17^+^ cells and CD4^+^Ets-1^+^cells in thyroid section **(A, B),** the staining method was the same as in **Figures 3C** and **4D**.

To detect the degree of thyroiditis after thyroid injection, we assessed the lymphocyte infiltration score of the thyroid and the serum titer of TgAb. The thyroiditis score in the LV-sponge group was significantly lower than that in the LV-ctrl group (*P* < 0.05), as shown in **Figure 6A**. Moreover, the serum TgAb level in the LV-sponge group decreased significantly compared with that in the LV-ctrl group (*P < 0.05, **Figure 6B**), consistent with the inhibition of miR-326 in the tail vein injection groups. In addition, the results of immunofluorescence indicated that, compared with the control group, CD4^+^IL-17a^+^ cells had a downward trend in LV-sponge groups, while CD4^+^Ets-1^+^ cells were increased significantly in the LV-sponge group (**Figures 7A, B**).

### Comparison of Two Injection Methods

**Figure 8:**
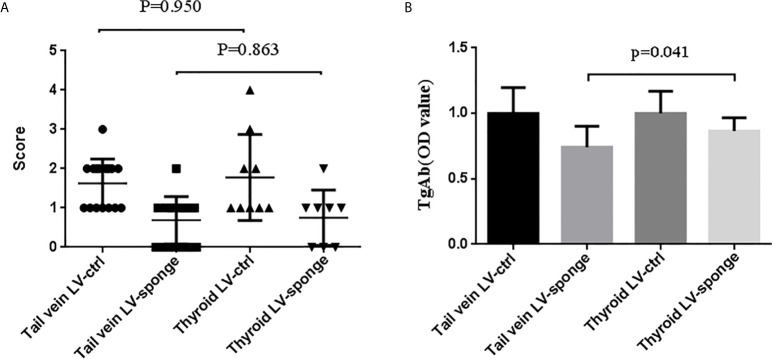
Comparison of the thyroiditis score **(A)** and TgAb titers **(B)** of NOD.H-2h4 mice that received tail vein injection and thyroid injection to evaluate the effect of injection intervention on thyroiditis.

We compared the inhibitory effects of these two injection methods on thyroiditis. We found that the tail vein LV-sponge group had a much lower TgAb level of thyroiditis compared with the thyroid LV-sponge group (*P* = 0.041). The lymphocyte infiltration inflammatory score of the thyroid was not significantly different (*P* = 0.863), as shown in **Figures 8A, B**. The results suggest that tail vein injection may achieve a better intervention effect.

## Discussion

MiRNAs have emerging roles in the regulation of a variety of diseases including autoimmune diseases. In systemic sclerosis (SSc), scholars have found that microRNA-27a-3p targeting sFRP-1, a negative regulator of Wnt signaling, in dermal fibroblasts mediates fibrosis regression (25). MiR-210 was reported to be increased and to regulate Th17 and Th1 cells by inhibiting the expression of STAT6 and LYN in psoriasis (26). Honardoost MA et al. found that miR-326 was increased in elapsing phase of multiple sclerosis patients compared with remitting phase (27). Another study found that in antibody-positive type 1 diabetes mellitus (T1DM) patients, the expression level of miR-326 significantly increased (28). In our previous study, we found that miR-326 increased significantly in HT patients and a mouse model of AIT (22, 29). These studies suggested that miR-326 plays an important role in autoimmune diseases. In autoimmunity, CD4^+^T cell is one of the most important players (30, 31). In the inflamed joints of RA patients, the synovium is highly infiltrated by CD4^+^ T cells, and Th1, Th17, and Treg cells are important and coexist with stromal cells in the microenvironment of the inflamed joint (32, 33). In our previous study, we found that Th17 cells were increased and Treg cells were decreased in AIT pathogenesis (22, 29). In our *in vitro* experiments, it was found that Th17 cells were decreased in the downregulation of miR-326 level by using siRNA (22). This study further carried out miR-326 *in vivo* intervention experiment by administering a lentivirus sponge (LV-sponge, mmu-miR-326 sponge, which inhibits the activity of endogenous miR-326) through tail vein and local thyroid injection to further explore the mechanism of the regulation of the expression of CD4^+^ T cells *in vivo*. The sponge is designed as a miRNA decoy target and is an effective and specific inhibitor of miRNA, which is widely used in the study of disease pathogenesis (34). In our study, we explored whether miR-326 inhibition by lentivirus-mediated silencing could affect the balance of different T cell subtypes and B cell subsets in mice. We found that the counts of T cells, B cells, Th cells, and Tc cells in the LV-sponge groups were not different between the control group and the LV-control groups. Next, we explored the inflammation level of mouse thyroiditis. We found that the scores of thyroid inflammation and serum TgAb titers in the LV-sponge groups decreased significantly compared with those in the LV-control groups, especially in the prophylactic LV-sponge group. These results indicated that the level of thyroid inflammation in mice decreased by inhibiting the activity of miR-326 *in vivo*, and prophylactic treatments were more effective than treatments after the beginning of thyroid inflammation. In order to explore the changes of CD4+T cell subsets after miR-326 inhibition, the counts of Th1, Th2, Th17, and Treg cells in the spleen were assessed by flow cytometry. Th17 cell counts were found to decrease while the Treg cell counts increased in the LV-sponge groups, and the increase of Treg was significant in the prophylactic LV-sponge group, while the Th1 and Th2 cell counts were not significantly different among the groups. What’s more, we found that Th17/Treg ratio decreased in the LV-sponge groups, especially in the prophylactic LV-sponge group. Th17-related cytokines (IL-22), transcription factors (Rorgt), and surface receptors (CCR6 and IL-23r) were also significantly decreased in the LV-sponge groups. In addition, thyroid infiltrated CD4^+^IL-17a^+^ cells were decreased *in situ* in LV-sponge groups. Moreover, serum level of inflammation promoting cytokine of IL-17a was in decreasing trend, while that of anti-inflammatory cytokine of IL-10 was increased in the prophylactic LV-sponge group. IL-17a/IL-10 ratio was decreased significantly in LV-sponge group. *In vivo* intervention of miR-326 can reduce the number of pro-inflammatory Th17 cells, increase the number of anti-inflammatory Treg cells, reduce the Th17/Treg ratio, and promote the recovery of autoimmune thyroiditis inflammation. Our previous research had showed that Ets-1 protein decreased significantly in the spleen of AIT mice and was negatively correlated with miR-326 and IL-17. The results are consistent with those in HT patients (unpublished data). These results suggest that miR-326 may regulate Th17 cells by targeting Ets-1 protein at the translational level in AIT, which was further proven by using siRNA intervention *in vitro* (22), but we didn’t explore the changes of Treg at the time. Some researchers found that Ets-1 deficiency resulted in the decrease of Foxp3 mRNA in CD4^+^CD25^+^ Tregs of SLE patients (35). In the present study, Ets-1 protein expression in spleen cells was found to be increased in LV-sponge groups. Moreover, thyroid infiltrated CD4^+^Ets-1^+^ cells were increased significantly in the LV-sponge group. Ets-1 protein was a functional target of miR-326 as had been suggested by several previous studies (36). Our results also suggest that miR-326 may target Ets-1 protein at the translational level, since the mRNA levels of Ets-1 were not different among groups. We speculated that inhibited activity of miR-326 leads to increased expression of Ets-1, which is further related to the differential of CD4^+^ T cell (the increased Treg and decreased Th17). A variety of miRNAs including miR-326 are involved in autoimmune thyroiditis. For instance, miR-142-5p was increased in HT thyroid gland and plays an important role in HT pathogenesis by targeting CLDN1 (37). Dorris ER et al. found miR-141 displayed significant downregulation of thyroiditis in HT patients, and they speculated that miR-141 may regulate TGF-*β* pathway contributing to autoimmune thyroiditis (38). In addition to miRNAs’ negative regulation of key master regulators, miRNA synergism, in which several miRNAs cooperate to regulate targets expressions, is an important regulation pathway. Our previous study also confirmed that elevated miR-326 levels regulate the IL-23/IL-23R/Th17 cell axis by targeting a disintegrin and metalloprotease 17 (ADAM17) in Hashimoto’s thyroiditis (29). Furthermore, a reduction in the lymphocyte infiltration inflammatory score of thyroid glands and TgAb titers was achieved in LV-sponge treated AIT mice in our study. Moreover, consistent with the results of the tail vein injection groups, in the LV-sponge thyroid injection group, the TgAb titers and lymphocyte infiltration inflammatory score of the thyroid glands were also decreased. In addition, we further compared the difference between the two injection methods in the intervention effect of thyroiditis. We found that tail vein injection may achieve a better intervention effect. We speculated that the tail vein injection could alleviate inflammation better in autoimmune diseases because it could be seen as a systemic immune regulation.

In NOD.H-2^h4^ mice, thyroid lesions start to develop in most mice 3–4 weeks after they are given NaI in drinking water, and thyroid lesions reach maximal severity 8–9 weeks after given NaI in drinking water (19). In our study, the therapeutic groups, the starting time of mice tail vein injection was followed by 6 weeks of iodine water feeding, while the beginning time of tail vein injection in the prophylactic group was the same as that of iodine water feeding. With iodine water intake for 6 weeks in the therapeutic groups, the thyroid lesions have formed, and related inflammatory factors such as Th17 cells have been elevated. On this basis, the intervention was to slow down further inflammatory process. In prophylactic LV-sponge group, intervention was given before thyroid damage; it had better and more lasting inhibitory effect. The experimental results also showed that the counts of Th17 cells in the prophylactic LV-sponge group are lower.

In conclusion, our study is the first to explore the targeted therapeutic and prophylactic effects of miR-326 *in vivo* by LV sponge. We confirmed that miR-326 contributes to autoimmune thyroiditis by targeting Ets-1 protein to regulate Th17/Treg balance in autoimmune thyroiditis, and tail vein injection may achieve a better intervention effect. However, we also realize that thyroiditis has not been completely inhibited, which indicates that the occurrence and development mechanism of AIT are complex, and miR-326 participates in only a part of it. What’s more, ADAM17, IL-23, and other transcription factors that may be related to miR-326 should also be studied, which is also the limitation of our study.

## Data Availability Statement

The raw data supporting the conclusions of this article will be made available by the authors, without undue reservation.

## Ethics Statement

The animal study was reviewed and approved by Animal Ethics Committee of China Medical University.

## Author Contributions

YL, NZ, ZS, and WT contributed to the concept and design of this study. NZ and ZW completed the mice experimental operation *in vivo*. NZ, XC, SW, and CF carried out the statistical analysis. NZ wrote the first draft of the manuscript. YL reviewed the final manuscript. All authors contributed to the article and approved the submitted version.

## Funding

The present work was supported by the Chinese National Natural Science Foundation (Grant nos. 81471003 and 81770784).

## Conflict of Interest

The authors declare that the research was conducted in the absence of any commercial or financial relationships that could be construed as a potential conflict of interest.

## References

[1] Caturegli P De Remigis A Rose NR. Hashimoto Thyroiditis: Clinical and Diagnostic Criteria. Autoimmun Rev (2014) 13(4-5):391–7. 10.1016/j.autrev.2014.01.007 24434360

[2] Burek CL Rose NR. Autoimmune Thyroiditis and ROS. Autoimmun Rev (2008) 7(7):530–7. 10.1016/j.autrev.2008.04.006 18625441

[3] Teng X Shan Z Chen Y Lai Y Yu J Shan L. More Than Adequate Iodine Intake may Increase Subclinical Hypothyroidism and Autoimmune Thyroiditis: A Cross-Sectional Study Based on Two Chinese Communities With Different Iodine Intake Levels. Eur J Endocrinol (2011) 164(6):943–50. 10.1530/EJE-10-1041 21444648

[4] Luo Y Kawashima A Ishido Y Yoshihara A Oda K Hiroi N. Iodine Excess as an Environmental Risk Factor for Autoimmune Thyroid Disease. Int J Mol Sci (2014) 15(7):12895–912. 10.3390/ijms150712895 PMC4139880 25050783

[5] Teng X Shan Z Teng W Fan C Wang H Guo R. Experimental Study on the Effects of Chronic Iodine Excess on Thyroid Function, Structure, and Autoimmunity in Autoimmune-Prone NOD.H-2h4 Mice. Clin Exp Med (2009) 9(1):51–9. 10.1007/s10238-008-0014-0 18953634

[6] Vecchiatti S Guzzo M Caldini E Bisi H Longatto-Filho A Lin C. Iodine Increases and Predicts Incidence of Thyroiditis in NOD Mice: Histopathological and Ultrastructural Study. Exp Ther Med (2013) 5(2):603–7. 10.3892/etm.2012.826 PMC3570204 23408765

[7] Fallahi P Ferrari SM Ruffilli I Elia G Biricotti M Vita R. The Association of Other Autoimmune Diseases in Patients With Autoimmune Thyroiditis: Review of the Literature and Report of a Large Series of Patients. Autoimmun Rev (2016) 15(12):1125–8. 10.1016/j.autrev.2016.09.009 27639841

[8] Noack M Miossec P. Th17 and Regulatory T Cell Balance in Autoimmune and Inflammatory Diseases. Autoimmun Rev (2014) 13(6):668–77. 10.1016/j.autrev.2013.12.004 24418308

[9] Fasching P Stradner M Graninger W Dejaco C Fessler J. Therapeutic Potential of Targeting the Th17/Treg Axis in Autoimmune Disorders. Mol (Basel Switzerland) (2017) 22(1). 10.3390/molecules22010134 PMC6155880 28098832

[10] Lee G. The Balance of Th17 Versus Treg Cells in Autoimmunity. Int J Mol Sci (2018) 19(3). 10.3390/ijms19030730 PMC5877591 29510522

[11] Shao S Yu X Shen L. Autoimmune Thyroid Diseases and Th17/Treg Lymphocytes. Life Sci (2018) 192:160–5. 10.1016/j.lfs.2017.11.026 29158050

[12] Wang D Huang S Yuan X Liang J Xu R Yao G. The Regulation of the Treg/Th17 Balance by Mesenchymal Stem Cells in Human Systemic Lupus Erythematosus. Cell Mol Immunol (2017) 14(5):423–31. 10.1038/cmi.2015.89 PMC5423084 26435067

[13] An N Chen Y Wang C Yang C Wu Z Xue J. Chloroquine Autophagic Inhibition Rebalances Th17/Treg-Mediated Immunity and Ameliorates Systemic Lupus Erythematosus. Cell Physiol Biochem Int J Exp Cell Physiol Biochem Pharmacol (2017) 44(1):412–22. 10.1159/000484955 29141242

[14] Li Q Wang B Mu K Zhang J. The Pathogenesis of Thyroid Autoimmune Diseases: New T Lymphocytes - Cytokines Circuits Beyond the Th1-Th2 Paradigm. J Cell Physiol (2019) 234(3):2204–16. 10.1002/jcp.27180 30246383

[15] Li C Yuan J Zhu Y Yang X Wang Q Xu J. Imbalance of Th17/Treg in Different Subtypes of Autoimmune Thyroid Diseases. Cell Physiol Biochem Int J Exp Cell Physiol Biochem Pharmacol (2016) 40:245–52. 10.1159/000452541 27855396

[16] Moisan J Grenningloh R Bettelli E Oukka M Ho IC. Ets-1 is a Negative Regulator of Th17 Differentiation. J Exp Med (2007) 204(12):2825–35. 10.1084/jem.20070994 PMC2118518 17967903

[17] Xiang N Li X Li X Wang G Tao J Pan H. Expression of Ets-1 and FOXP3 mRNA in CD4(+)CD25 (+) T Regulatory Cells From Patients With Systemic Lupus Erythematosus. Clin Exp Med (2014) 14(4):375–81. 10.1007/s10238-013-0263-4 24221578

[18] Podolin PL Pressey A DeLarato NH Fischer PA Peterson LB Wicker LS. I-E+ Nonobese Diabetic Mice Develop Insulitis and Diabetes. J Exp Med (1993) 178(3):793–803. 10.1084/jem.178.3.793 8350054 PMC2191185

[19] Braley-Mullen H Yu S. Nod.H-2h4 Mice: An Important and Underutilized Animal Model of Autoimmune Thyroiditis and Sjogren’s Syndrome. Adv Immunol (2015) 126:1–43. 10.1016/bs.ai.2014.11.001 25727287

[20] Vladutiu AO Rose NR. Autoimmune Murine Thyroiditis Relation to Histocompatibility (H-2) Type. Science (1971) 174(4014):1137–9. 10.1126/science.174.4014.1137 5133731

[21] Braley-Mullen H Sharp GC Medling B Tang H. Spontaneous Autoimmune Thyroiditis in NOD.H-2h4 Mice. J Autoimmun (1999) 12(3):157–65. 10.1006/jaut.1999.0272 10222025

[22] Zhao N Zou H Qin J Fan C Liu Y Wang S. MicroRNA-326 Contributes to Autoimmune Thyroiditis by Targeting the Ets-1 Protein. Endocrine (2018) 59(1):120–9. 10.1007/s12020-017-1465-4 29181619

[23] Nagayama Y Horie I Saitoh O Nakahara M Abiru N. CD4+CD25+ Naturally Occurring Regulatory T Cells and Not Lymphopenia Play a Role in the Pathogenesis of Iodide-Induced Autoimmune Thyroiditis in NOD-H2h4 Mice. J Autoimmun (2007) 29(2-3):195–202. 10.1016/j.jaut.2007.07.008 17826032

[24] Horie I Abiru N Nagayama Y Kuriya G Saitoh O Ichikawa T. T Helper Type 17 Immune Response Plays an Indispensable Role for Development of Iodine-Induced Autoimmune Thyroiditis in Nonobese diabetic-H2h4 Mice. Endocrinology (2009) 150(11):5135–42. 10.1210/en.2009-0434 19797122

[25] Henderson J Wilkinson S Przyborski S Stratton R O’Reilly S. microRNA27a-3p Mediates Reduction of the Wnt Antagonist sFRP-1 in Systemic Sclerosis. Epigenetics (2020) 4:1–10. 10.1080/15592294.2020.1827715 PMC8216176 32965161

[26] Wu R Zeng J Yuan J Deng X Huang Y Chen L. MicroRNA-210 Overexpression Promotes Psoriasis-Like Inflammation by Inducing Th1 and Th17 Cell Differentiation. J Clin Invest (2018) 128(6):2551–68. 10.1172/JCI97426 PMC5983326 29757188

[27] Honardoost M Kiani-Esfahani A Ghaedi K Etemadifar M Salehi M. miR-326 and miR-26a, Two Potential Markers for Diagnosis of Relapse and Remission Phases in Patient With Relapsing-Remitting Multiple Sclerosis. Gene (2014) 544(2):128–33. 10.1016/j.gene.2014.04.069 24792898

[28] Sebastiani G Grieco F Spagnuolo I Galleri L Cataldo D Dotta F. Increased Expression of microRNA miR-326 in Type 1 Diabetic Patients With Ongoing Islet Autoimmunity. Diabetes/metabolism Res Rev (2011) 27(8):862–6. 10.1002/dmrr.1262 22069274

[29] Liu Y Cui X Wang S Liu J Zhao N Huang M. Elevated MicroRNA-326 Levels Regulate the IL-23/IL-23R/Th17 Cell Axis in Hashimoto’s Thyroiditis by Targeting a Disintegrin and Metalloprotease 17. Thyroid Off J Am Thyroid Assoc (2020) 30(9). 10.1089/thy.2019.0552 32204685

[30] Dittel B. Cd4 T Cells: Balancing the Coming and Going of Autoimmune-Mediated Inflammation in the CNS. Brain Behav Immun (2008) 22(4):421–30. 10.1016/j.bbi.2007.11.010 PMC2376206 18207698

[31] Krebs CF Kapffer S Paust HJ Schmidt T Bennstein SB Peters A. MicroRNA-155 Drives TH17 Immune Response and Tissue Injury in Experimental Crescentic GN. J Am Soc Nephrol (2013) 24(12):1955–65. 10.1681/ASN.2013020130 PMC3839549 23949802

[32] Weyand CM Goronzy JJ. Immunometabolism in Early and Late Stages of Rheumatoid Arthritis. Nat Rev Rheumatol (2017) 13(5):291–301. 10.1038/nrrheum.2017.49 28360422 PMC6820517

[33] Okamoto H Cujec TP Yamanaka H Kamatani N. Molecular Aspects of Rheumatoid Arthritis: Role of Transcription Factors. FEBS J (2008) 275(18):4463–70. 10.1111/j.1742-4658.2008.06582.x 18662303

[34] Ebert M Neilson J Sharp P. MicroRNA Sponges: Competitive Inhibitors of Small RNAs in Mammalian Cells. Nat Methods (2007) 4(9):721–6. 10.1038/nmeth1079 PMC3857099 17694064

[35] Sun X Tao J Xiang N Li X Wang G Fang X. Negative Correlation Between miR-326 and Ets-1 in Regulatory T Cells From New-Onset SLE Patients. Inflammation (2016) 39(2):822–9. 10.1007/s10753-016-0312-8 26861134

[36] Du C Liu C Kang J Zhao G Ye Z Huang S. MicroRNA miR-326 Regulates TH-17 Differentiation and is Associated With the Pathogenesis of Multiple Sclerosis. Nat Immunol (2009) 10(12):1252–9. 10.1038/ni.1798 19838199

[37] Zhu J Zhang Y Zhang W Zhang W Fan L Wang L. MicroRNA-142-5p Contributes to Hashimoto’s Thyroiditis by Targeting CLDN1. J Trans Med (2016) 14(1):166. 10.1186/s12967-016-0917-6 PMC4898455 27277258

[38] Dorris E Smyth P O’Leary J Sheils O. Mir141 Expression Differentiates Hashimoto Thyroiditis From PTC and Benign Thyrocytes in Irish Archival Thyroid Tissues. Front Endocrinol (2012) 3. 10.3389/fendo.2012.00102 PMC3432448 22969748

